# *In vitro* cytotoxicity activity of novel Schiff base ligand–lanthanide complexes

**DOI:** 10.1038/s41598-018-21366-1

**Published:** 2018-02-14

**Authors:** Kavitha Andiappan, Anandhavelu Sanmugam, Easwaramoorthy Deivanayagam, K. Karuppasamy, Hyun-Seok Kim, Dhanasekaran Vikraman

**Affiliations:** 1 Department of Chemistry (S & H), Vel Tech Multi Tech, Chennai, 600062 India; 2 Department of Chemistry, B.S Abdur Rahman University, Vandalur, Chennai 600048 India; 3 Division of Electronics and Electrical Engineering, Dongguk University-Seoul, Seoul, 04620 Republic of Korea

## Abstract

A Schiff base ligand (SBL), N^2^, N^3^-bis (anthracen-9-ylmethylene) pyridine-2, 3-diamine, was synthesized through the condensation of 2,6-diaminopyridine and anthracene-9-carbaldehyde using a 1:2 ratio. ^1^H NMR spectra confirmed the observation of non-involvement aromatic carboxylic proton in SBL. A novel series of lanthanide (i.e., praseodymium (Pr), erbium (Er), and ytterbium (Yb))-based SBL metal complexes was successfully synthesized, and their functional groups were elaborately demonstrated using UV–visible, Fourier transform infrared (FT-IR), and fluorescence spectroscopy analyses. FT-IR spectral studies revealed that SBL behaved as a bidentate ligand and it was structured with metal ions by the two azomethine nitrogens. The synthesized SBL-based metal complexes were elaborately performed for cytotoxicity activity versus Vero, human breast cancer (MCF7), and cervical (HeLa) anticancer cell lines.

## Introduction

After the innovative success of cisplatin as a medically recognized antitumor drug, medicinal chemists began interdisciplinary research on metal complexes for interaction with DNA/RNA, biomolecules, and proteins as antitumor drugs^1,2^. On the other hand, the use of platinum (Pt) metal-based cisplatin drug in medicinal purposes causes numerous side effects, which remains a challenge to overcome to prepare efficient anticancer drugs^3–5^. Medicinal inorganic chemistry offers an extensive possibility for the design of novel drugs based on the coordination and redox properties of metal complexes to fight against cancer^6,7^. Currently, various metal complexes, including copper, lanthanum, and ruthenium, complexes, are considered as the most capable replacements for classical cisplatin-type drugs^7–17^.

For decades, Schiff bases have been considered important ligands due to their coordination chemistry, and they can be easily prepared and linked with a different kind of metal ions^18^. Due to the existence of an amine group, analogous to the natural biological systems, Schiff bases show a crucial role in observing the conversion mechanism and racemization reaction in biological systems^19–25^. Schiff bases have been used for various essential biological activities including antitumor, anti HIV, antibacterial, antifertility activities, antimosquito larval, antiinflammatory, and anticancer^26–33^. Gokhale *et al*.^34^ examined the activity of the ligand-based distorted square planar copper(II) complex, cis-[dichloro (N1-(2-benzyloxybenzylidene)pyridine-2-carboxamidrazone) copper(II)], against the human breast cancer cell line MCF-7 using the MTT method. Among the various metal complexes, lanthanide-based metal complexes have been deeply examined owing to their low poisonousness and high biological activities after bonding with ligands. In recent years, the research community has shown great interest in the synthesis of lanthanide-based complexes due to their applicability in DNA interaction and anticancer activity^35–37^. Wang *et al*.^38^ have reported a Schiff base La(III) complex prepared from kaempferol and diethylenetriamine interaction with DNA by intercalation mode and observed stronger La(III) complex binding and cleaving capacities with DNA than ligands by *in vitro* cytotoxic behaviors against HepG-2 cell lines and HL-60 cells (the human leukocytoma). Zaho *et al*.^39^ have described a La(III) (N′N-bis-(1-carboxy-2-methylpropyl)-1,10-phenanthroline-2,9-dimethanamine) complex for antitumor activity *in vitro* against KB (human nasopharyngeal carcinoma) cells, BGC-823 (human stomach carcinoma) cells, Bel-7402 (human liver carcinoma) cells, HCT-8 (human coloadenocarcinoma) cells, and HL-60 cells. Neelima *et al*.^9^ have screened the Schiff base ligand (SBL) and its La(III) complex, 2,3-dihydro-1*H*-indolo-[2,3-*b*]-phenazin-4(5*H*)-ylidene)benzothiazole–2-amine (L^1^), for anticancer activity against PC-3 cell lines (human prostate carcinoma) and found the reduction of cell viability of prostate cancer cell lines in a dose-dependent manner.

In the present investigation, a bidentate SBL and its novel lanthanide metal(III) complexes (praseodymium, erbium, and ytterbium) were successfully synthesized by simple one-pot chemical synthesis and plausibly characterized by analytical techniques including UV-visible (UV-vis), Fourier transform infrared (FT-IR), and fluorescence spectroscopy. In addition, their cytotoxicity effects on Vero, HeLa, and MCF7 cancer cell lines *in vitro* were demonstrated in detail.

## Results and Discussion

**Figure 1:**
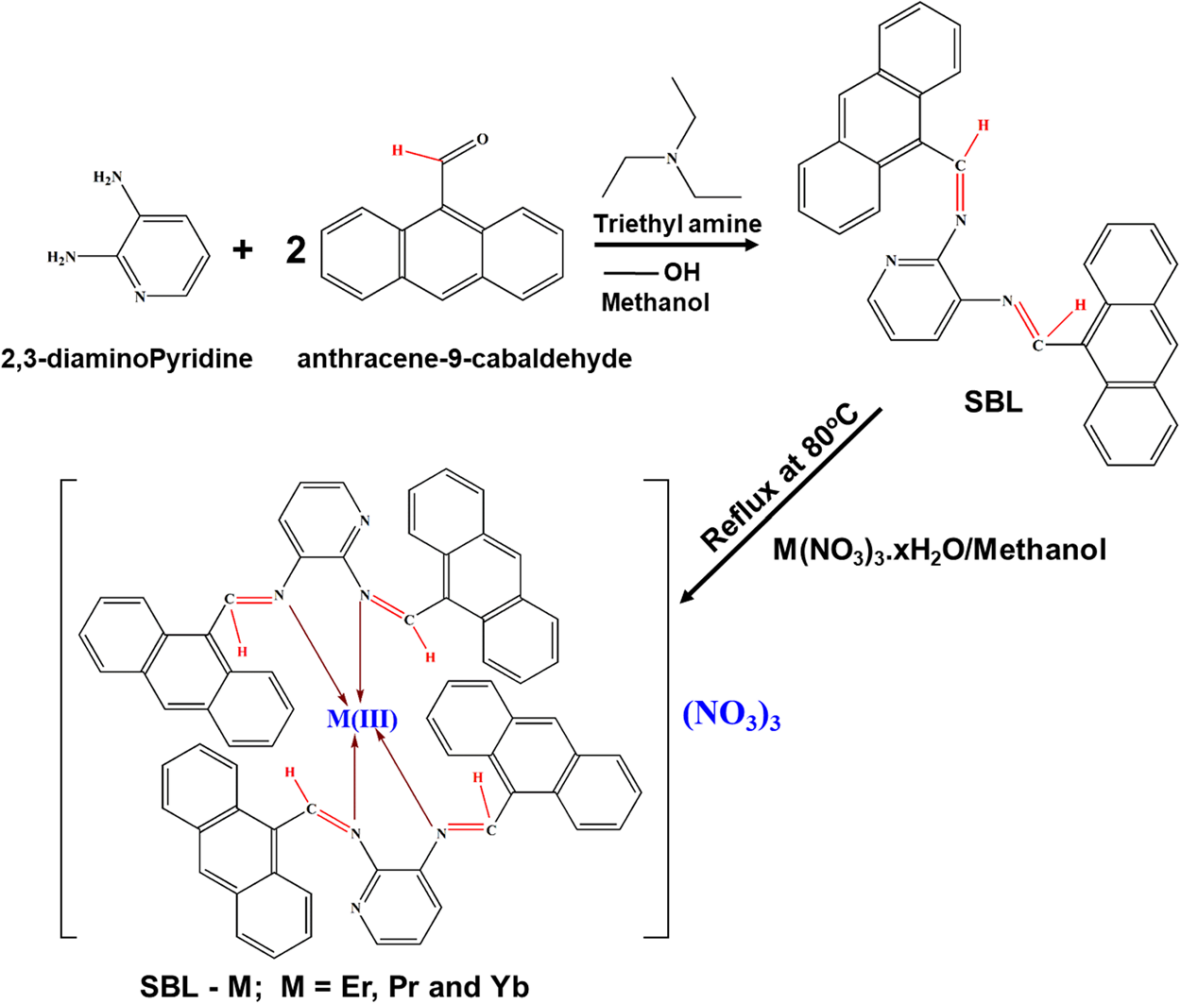
Schematic methodology of SBL and its metal complex preparation.

An SBL, N^2^, N^3^-bis (anthracen-9-ylmethylene) pyridine-2, 3-diamine, was synthesized through the condensation of 2,6-diaminopyridine and anthracene-9-carbaldehyde using a 1:2 ratio. The schematic representation of SBL synthesis is given in Fig. 1. Figure S1 (a) shows a ^1^H NMR spectrum of the SBL complex that exhibits a specific signal at 12.23 ppm due to NH protons, and the signal in the range of 8.0–8.7 ppm is attributed to azomethine protons. A signal is also observed in the 6.63–8.29 ppm region due to aromatic protons^40,41^. From a ^13^C NMR spectrum (Figure S1b), SBL exhibits a specific signal at 165.37 ppm due to azomethine carbon^42,43^. It also shows a signal in the range of 126–132 ppm that corresponds to the aromatic carbons^44,45^. The observed NMR results confirmed the successful formation of the SBL complex. Further, a novel series of lanthanide (i.e., praseodymium (Pr), erbium (Er) and ytterbium (Yb))-based SBL metal complexes was successfully synthesized by simple one-pot chemical synthesis. The detailed experimental procedures used to prepare the SBL-based metal complexes are elaborated in the experimental part. The preparation scheme for the Schiff base ligand–Pr (SBLPr), Schiff base ligand–Er (SBLEr), and Schiff base ligand–Yb (SBLYb) metal complexes is shown in Fig. 1.

### Functional properties analyses of SBL and its metal complexes

**Figure 2:**
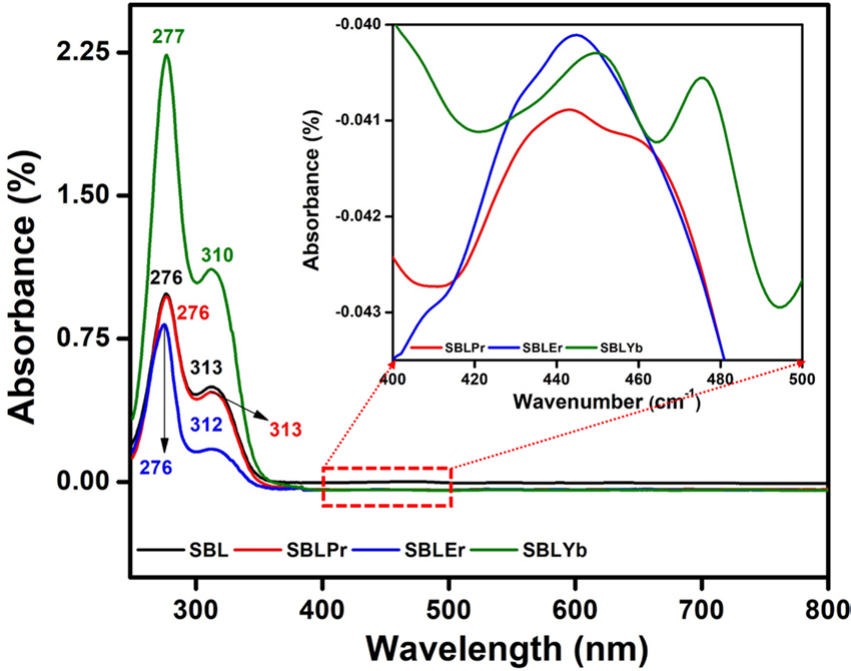
UV-vis spectra of SBL and its metal complexes in range of 250–800 nm.

Electronic spectral studies are important tools to identify the structure of ligands and their metal complexes, and they are also used for examining the stereochemistry of metal ions in the complexes based on their peak locations and the digit of d–d transition peaks. The UV-vis spectra of the SBL and its metal complexes (i.e., SBLPr, SBLEr, and SBLYb) were recorded at room temperature using methanol as the solvent. Figure 2 shows the UV-vis electronic spectra for the SBL and its metal complexes. The UV-vis spectra of the SBL revealed two strong absorption bands of intra-ligand charge transfer transition at 276 and 313 nm, which can be attributed to the π-π* transition and n-π* transitions of the carbonyl group of anthracene-9-carbaldehyde, respectively, and a slight shift for SBLEr and SBLYb, except SBLPr, metal complexes. In addition, the electronic absorption spectra of metal complexes, such as SBLPr, SBLEr, and SBLYb, revealed a broad band in the region between 420 and 480 nm, particularly at 442, 445, and 450 nm, respectively. These bands are attributed to ^2^B_1g_ → ^2^A_1g_ transition, which is characteristic of the distorted square planar geometry of Pr, Er, and Yb ions. The band positions of the absorption maxima, band assignments, and geometry of the complexes are tabulated in supporting information Table S1. The obtained results are in agreement with earlier results^46,47^.

**Figure 3:**
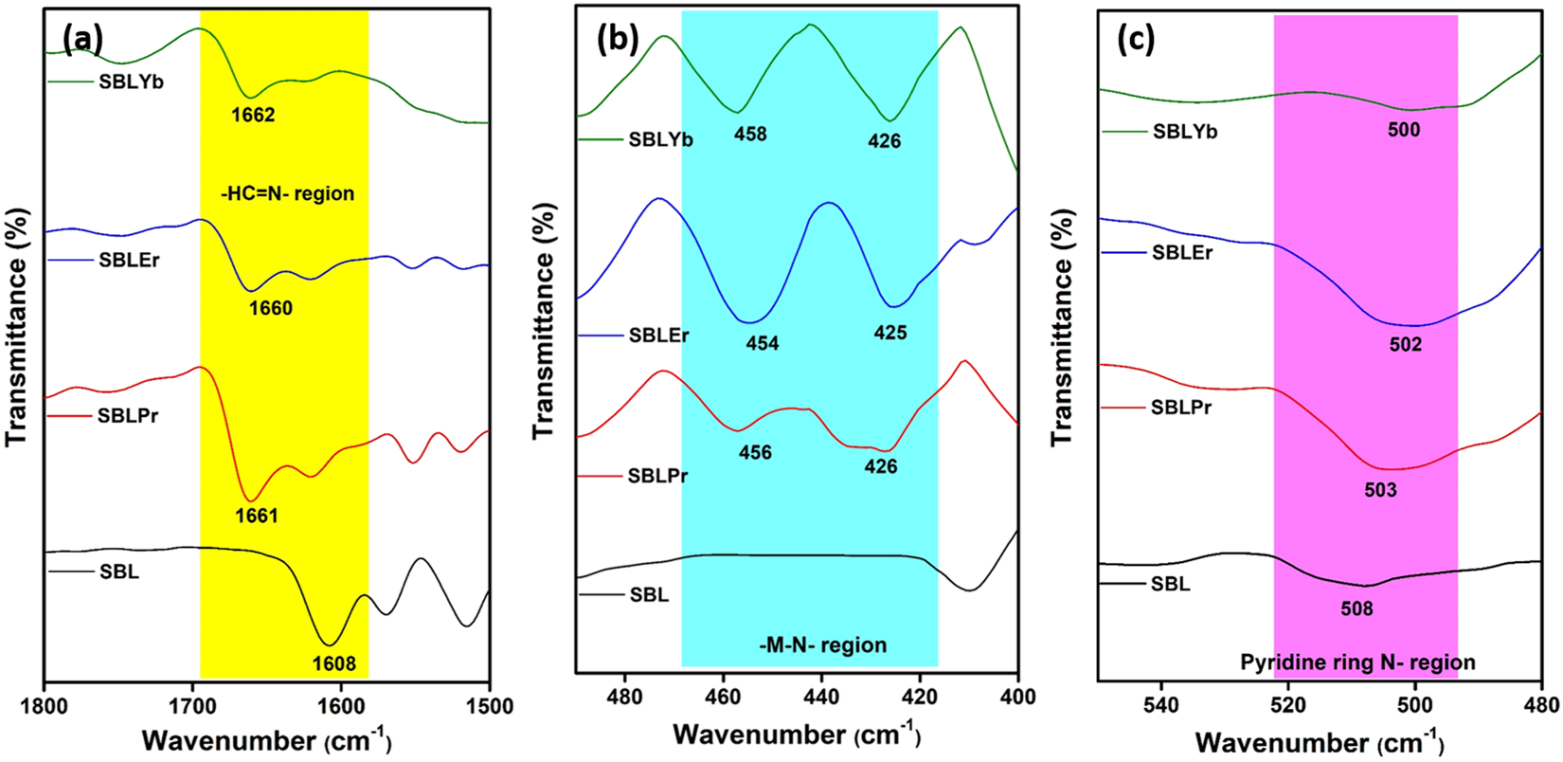
FT-IR spectra of SBL and its metal complexes. (**a**) Azomethine group (ν-_HC=N_) region, (**b**) -M-N-region, and (**c**) Pyridine ring N-region.

To identify the bonding mode as well as the complexation behavior of the prepared SBL and its metal complexes, the FT-IR spectral characteristics were recorded for SBL and its metal complexes. For 2,3-diaminopyridine, the characteristic vibrational peaks were reported at 3390, 3309, 2883, 1637, 1577, 1184, 1120, and 1064 cm^−1^ corresponding to N-H asymmetric stretching, N-H symmetric stretching, C-H aromatic symmetric stretching, N-H stretching of the pyridine ring, C=C stretching, C-N stretching, C-H in-plane bending, and C-O stretching, respectively^48^. Moreover, the anthracene-9-carbaldehyde characteristic vibrational peaks were reported at 2924–2854, 1682, and 1120 cm^−1^ corresponding to aromatic C-H stretching, C=O stretching, and C=C aromatic ring stretching, respectively. The FT-IR spectra of the SBL and its metal complexes are displayed in Fig. 3. The formation of the SBL is confirmed by the presence of a strong absorption band at 1608 cm^−1^, which represents the azomethine group (ν-_HC=N_), as shown in Fig. 3(a). The addition of metal nitrate salts to the SBL affects the structural organization of metal complexes and shifts the wavenumber regions toward higher wavenumbers^49,50^. For instance, the absorption bands of the metal complexes, SBLPr, SBLEr, and SBLYb, are shifted to higher wavenumbers than those of the SBL (i.e., 1661, 1660, and 1662 cm^−1^, respectively) in the spectra. This suggests that the azomethine nitrogen of SBL is involved in chelation (Fig. 3a) and hence that complexation takes place through the N-atom of the azomethine group. The coordination between the metal ion and N-atom of the azomethine group is further confirmed by analyzing the FT-IR spectra in the region between 520 and 400 cm^−1^. In this region, the FT-IR spectra of all the metal complexes show strong bands, which may be due to M-N symmetric and asymmetric stretching vibrations, as shown in Fig. 3(b). In addition, it is important to note that no significant changes are observed in the wavenumber region 550–480 cm^−1^, which indicates that the pyridine ring N-atom does not participate in binding or chelation with metal ions, as shown in Fig. 3(c). Correspondingly, all the prepared metal complexes (SBLPr, SBLEr, and SBLYb) show vibrational bands in the region between 1110 and 1120 cm^−1^, which corresponds to phenyl ring vibrations^51^. The other important vibrational peaks and their corresponding assignments are tabulated in supporting information Table S2. The molar conductance study was carried out using di-methyl sulfoxide (DMSO) as a solvent and their values are tabulated in supporting information Table S3. The observed values (100–128 ohm^−1^ cm^2^ mol^−1^) for the metal complexes indicates that the synthesized complexes are in electrolytic nature with M(III) ions and the nitrate ions are proposed to be present in the outside of coordination sphere. The observed results are in greatly concurrent with the earlier reports^52^.

**Figure 4:**
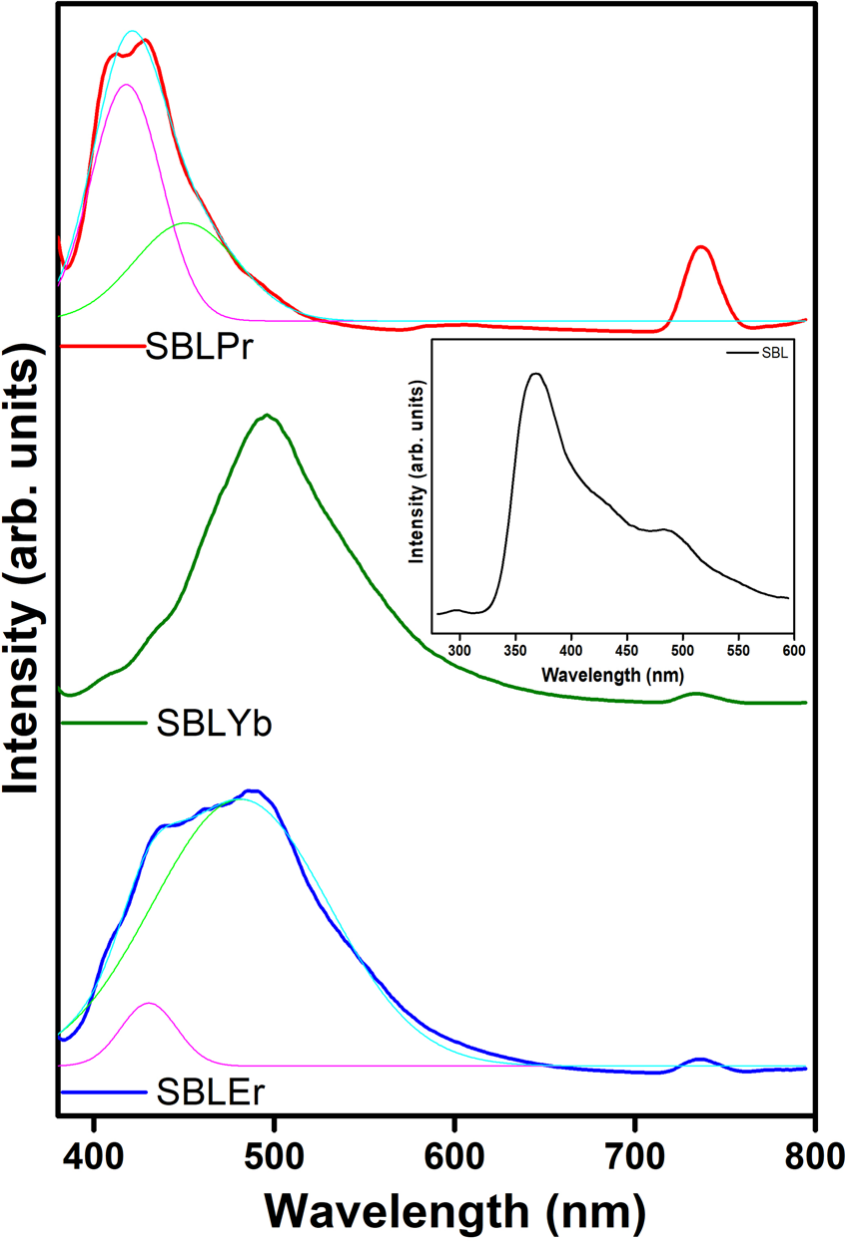
Fluorescence spectra of SBL metal complexes (Inset: fluorescence spectrum of SBL). The deconvoluted spectra included for SBLEr and SBLPr metal complexes were due to their combined broad spectral peak.

Synthesized metal complexes have excellent fluorescence behavior in nature, and their emission spectra were recorded at a 300-nm excitation wavelength. The SBL shows an intense peak at 369 nm and a weaker broad peak at 495 nm, which are attributed due to the π –π* transition, as shown in the inset of Fig. 4. For the SBLPr metal complex, a combined emission band of SBL is observed in the range of 380–490 nm due to the π –π* transition, which is displayed with its deconvoluted peaks, as shown in Fig. 4. The SBL peak broadening is increased with a higher wavelength shift due to the complex formation of Pr with SBL. For Pr, a sharp peak is observed at 736.5 nm in the SBLPr spectrum. In the case of the SBLYb complex, a sharp SBL peak is observed at 495 nm with a shoulder peak at 408 nm. A low-intensity broad peak is exhibited at 735.4 nm due to the fluorescence behavior of Yb. For the SBLEr metal complex, a combined emission band of SBL is observed in the range of 385–580 nm with a broad peak at 733.5 nm of Er, showing behavior similar to that of the SBLPr complex, as shown in Fig. 4. The fluorescence curves indicate that energy is transferred more efficiently from the lowest triplet state energy level (T_1_) of the ligand to the rare earth lowest resonance energy level (5D_4_)^53^. In an earlier report, a blue shift in the lanthanum(III) complex indicated the lanthanum ions have influenced the energy absorption of the complex system by the coordination with ligand^54,55^.

**Figure 5:**
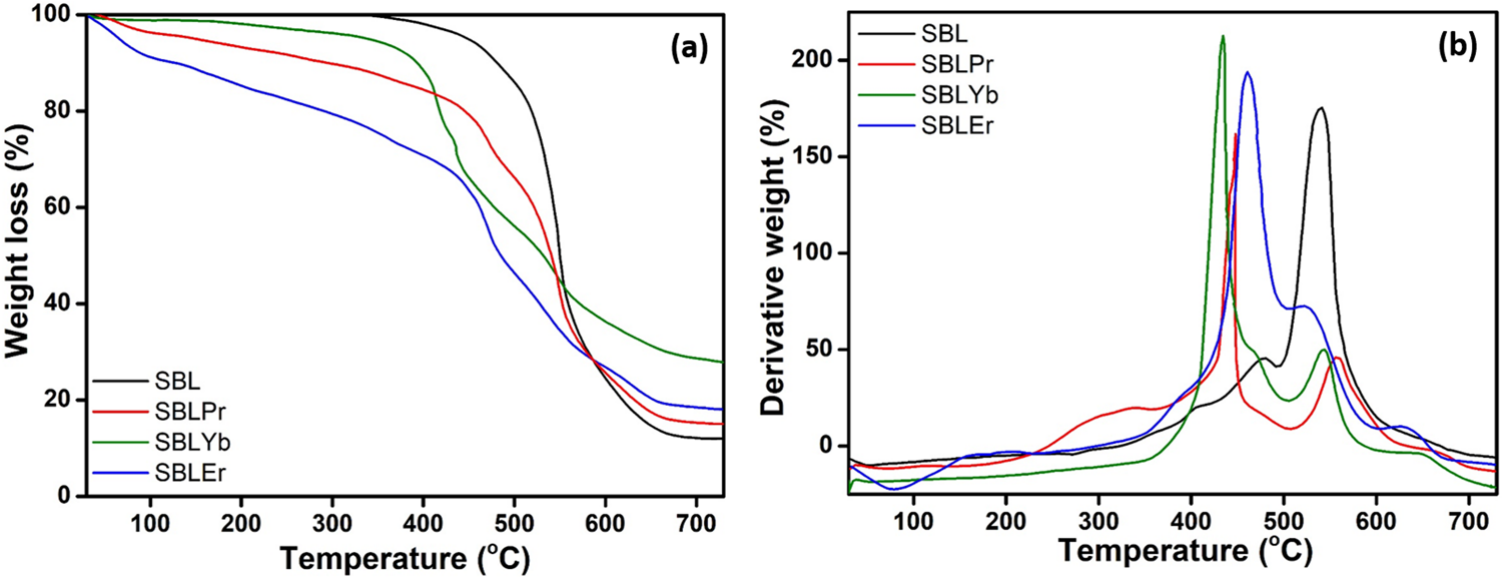
(**a**) TG and (**b**) DTA curves of SBL and its metal complexes.

**Figure 6:**
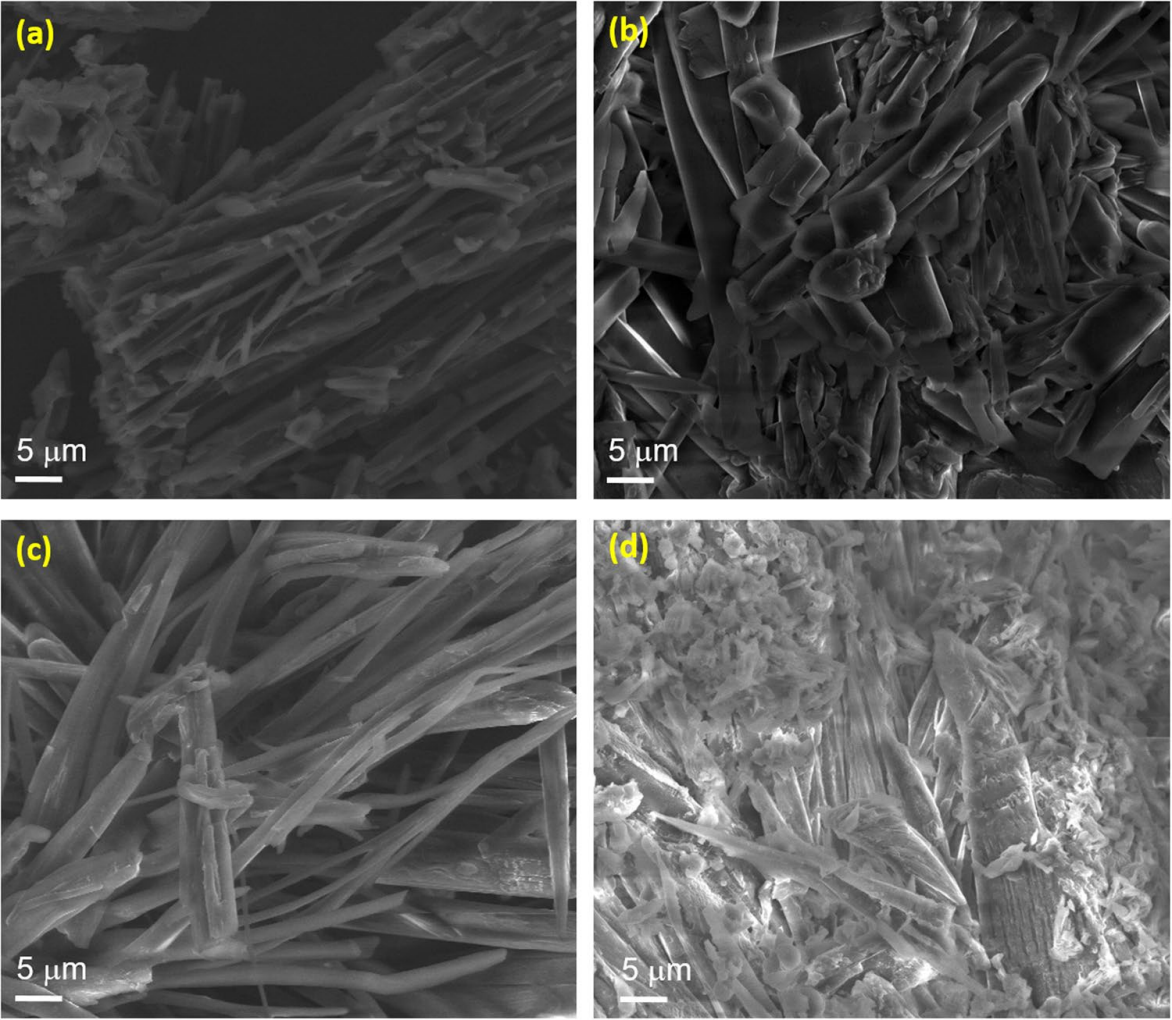
SEM micrographs of (**a**) SBL and (**b**) SBLPr, (**c**) SBLEr, and (**d**) SBLYb complexes.

Thermal stability as well as decomposition mechanisms for our SBL and its complexes of SBLEr, SBLPr, and SBLYb were analyzed by TG-DTG measurements under an inert atmosphere. According to the coordination chemistry, the inclusion of metal ions and alteration of the ligand’s functional groups could lead to thermal instability and alter its corresponding decomposition stages^56^. This investigation also demonstrated the thermal stability of SBL changes by the inclusion of rare earth metal ions in the SBL matrix. The precursor materials, such as 2,3-diaminopyridine (DAP) and anthrancene-9-carbaldehyde, showed one-step and two-step thermal decomposition with Td values of 287 and 368 °C, respectively, as reported earlier^57,58^. Figure 5(a) represents the thermograms of the SBL and the SBLEr, SBLPr, and SBLYb complexes. From the thermogram, the SBL shows two weight-loss stages. The earlier stage is ascribed to the decomposition of 2,3-diaminopyridine molecules, and the later stage is ascribed to the decomposition of anthracene-9-carbaldehyde. The residues obtained at 750 °C may be a carbon residue. Further, the mass loss happens at lower temperatures (237–345 °C) than that of precursor DAP, which indicates that the SBL is weaker than DAP. This may be due to the reduction in the number of primary amino groups of DAP after SBL formation. These results are in line with earlier reports^59–61^. Moreover, the TG curves of SBLPr, SBLYb, and SBLEr are exhibited with four-step degradation stages. The initial stage is due to the loss of physically adsorbed water molecules. The second stage is ascribed to the loss of free amino pyridine units. The third step is assigned to the deformation of the coordination bond between the azomethine nitrogen and metal ion and the decomposition of the condensed pyridine unit. The final step is attributed to the decomposition of anthracene carbaldehyde units. In addition, it is noticed from the thermogram that the thermal stability of the metal complexes seems to be lower than that of the SBL. This is due to the formation of thermally stable metal oxides, which results in the larger amount of residue weight loss in the complexes as compared to the SBL. The maximum amount of mass loss observed at higher temperatures in SBLYb than in the other complexes, which represents the ytterbium complex has better thermal stability compared with the erbium and praseodymium complexes^62,63^. The DTG curves of the SBL and its corresponding metal complexes are displayed in Fig. 5(b). The DTG curves of both the SBL and metal complexes suggest that thermal decay always involved exothermic decomposition. The endothermic peak positions are diverse for the SBL and its metal complexes which might be due to the difference in the complexation behavior between metal ions. The different weight losses of the SBL and its corresponding metal complexes obtained from TG-DTG analysis are tabulated in supporting information Table S4. The SEM micrographs of the SBL and its metal complexes (SBLPr, SBLEr, and SBLYb) are presented in Fig. 6 (a–d). The larger particles are formed by agglomeration, as predicted by morphological structure.

### Cytotoxicity behavior against Vero, MCF7, and HeLa cancer cell lines using SBLPr and SBLEr metal complexes

SBL-based metal complexes (SBLPr and SBLEr) are to be useful as anticancer drugs because of their stability, cytocompatibility, and flexible binding with biomolecules^64^. In recent times, the advancement of drug-delivery systems has been broadly attempted to produce the anticipated healing effect in patients with low opposing reactions^65^. In this work, the antiproliferative actions of SBLPr and SBLEr were examined in three different cancer cell lines (i.e., Vero, HeLa, and MCF7 cells) and evaluated by MTT assay.

**Figure 7:**
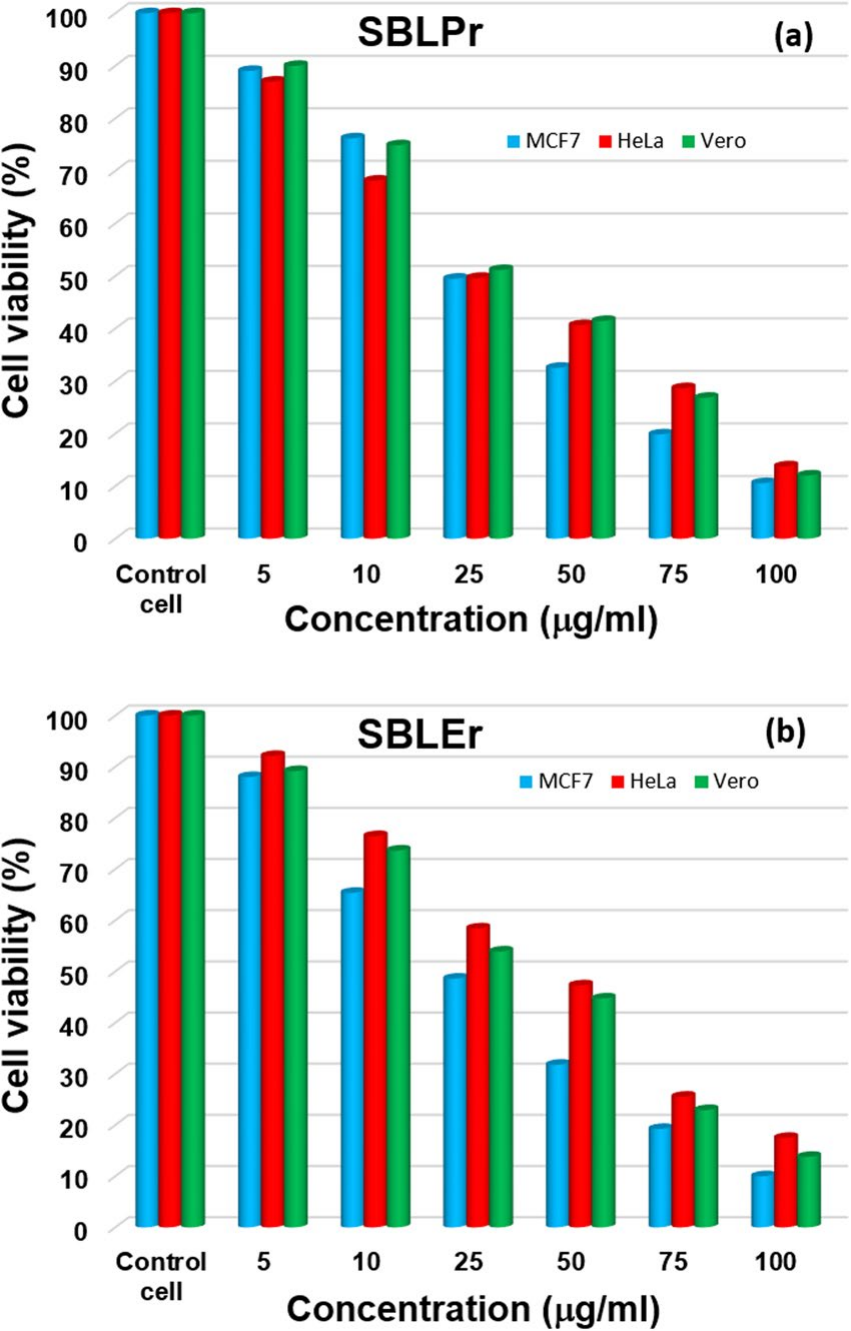
Cell viability of cancer cell lines (i.e., MCF7, HeLa, and Vero) against **(a)** SBLPr and **(b)** SBLEr metal complexes.

**Figure 8:**
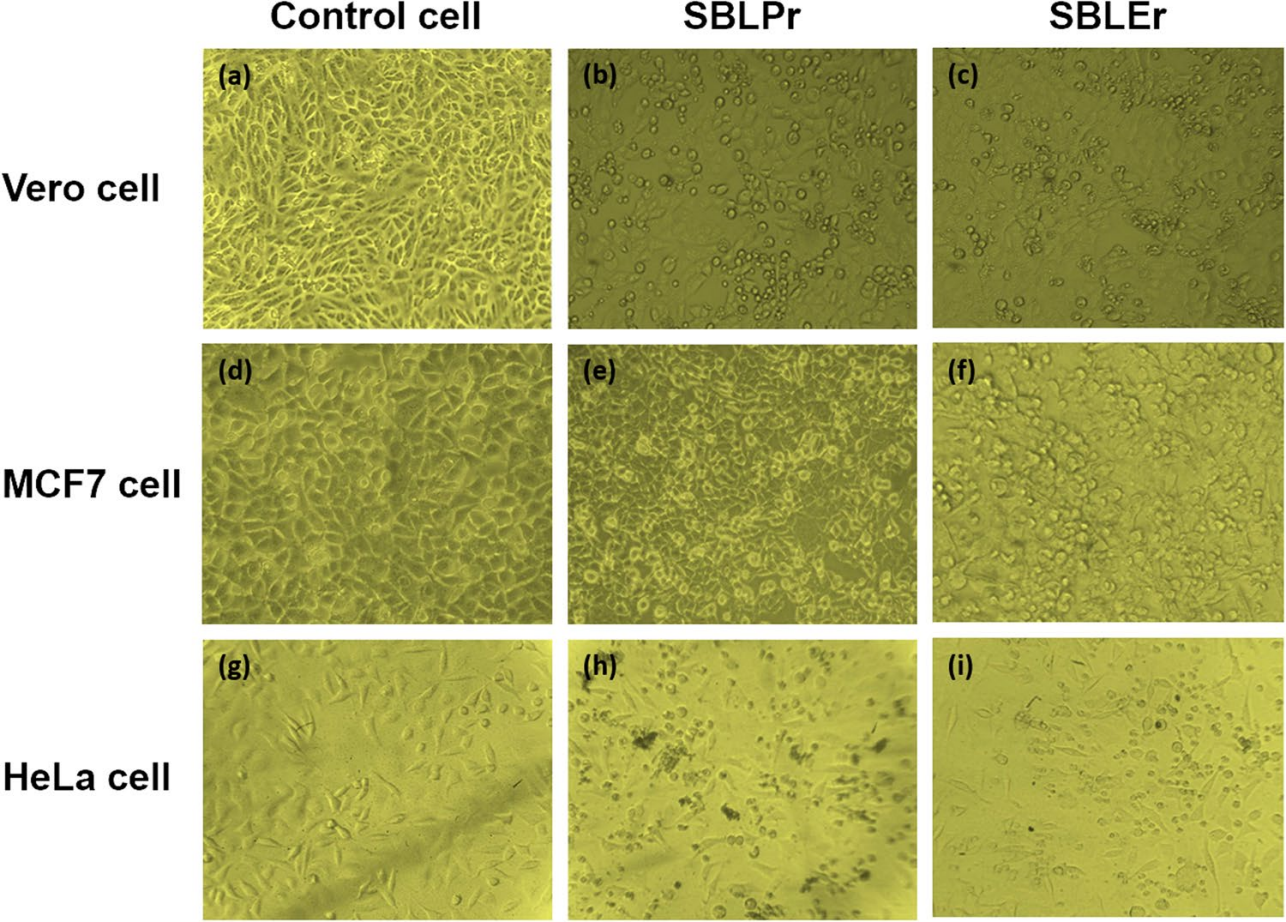
Morphological changes induced by SBLPr and SBLEr metal complexes using 25 μg/ml compared with control cancer cell lines.

Observations of the effect of synthesized SBLPr and SBLEr at different concentrations (i.e., 5, 10, 25, 50, 75, and 100 μg/ml complexes) on cell viability of Vero, MCF7, and HeLa cells were made at 24 h. The observed results explicated the cells’ viability at 85–90% for the concentrations up to 5 μg/ml, as shown in Fig. 7(a,b). As shown in Fig. 7, SBLPr and SBLEr efficiently induced apoptosis in Vero, MCF7, and HeLa cells in a dosage dependent manner. The SBLPr-tested Vero cells displayed a good biocompatibility compared to the complex of SBLEr, as shown in Fig. 7. The effective decrements in cell viability were noted in treated concentrations when compared to control cells. An earlier report revealed that the Vero cell lines at higher metal complex concentrations exposed substantial cell death^66,67^. Moreover, the biocompatibility characteristics of gold nanoparticles (AuNPs) were eventually performed versus Vero, HeLa, MCF7 and HeP-G2cell lines^68^. The Vero cells treated for 24 h with the respective IC_50_ concentration of SBLPr and SBLEr became rounded and began to shrink and lose their interaction with nearby cells. The morphological images shown in Fig. 8(a–c) confirmed the toxic effect of SBLPr and SBLEr (@25 μg/ml) samples against Vero cells compared with control cells.

For MCF7 and HeLa cells, the microscopic images of control cells and cells treated with 25 μg/ml SBLPr and SBLEr for 24 h are provided in Fig. 8(d–i). The MCF7 cells (Fig. 8d) consisted of irregular confluent combines with smooth-edged polygonal structures. The cells showed numerous cell surface protuberances. Under the controlled conditions, the untreated cancer cell lines (HeLa and MCF7) grew well with obvious skeletons. The cells appeared in a circular shape under an inverted microscope. After treatment with 25 μg/ml of SBLPr and SBLEr for 24 h, the cell volume was reduced and the cell density was decreased, as shown in Fig. 8. For the metal complex-treated cells, the metal (Pr) and (Er) ions from SBLPr and SBLEr, respectively, cooperated with phosphorus moieties in DNA, causing to control the DNA reproduction, which resulted in the loss of cell viability and cell death^54,69^. It has been validated that SBLPr and SBLEr are active metal complexes against human cancer cell lines^55,62^. Omar *et al*.^41^ have described the different concentrations of metal nanoparticles for cytotoxicity effects on MCF7 and HepG2 cancer cell lines. Likewise, Mohanan *et al*. have demonstrated the cytotoxicity behavior of crocin–metal nanoparticles against MCF7 cells after 24 and 48 h growth^40^. The observed results from the present study against MCF7 and HeLa cell lines treated with SBLPr and SBLEr (at 25 μg/ml) revealed cell death up to ~49% and ~42–51%, respectively. The cells treated with SBLPr and SBLEr underwent cell shrinkage, which is evidenced from Fig. 8. Moreover, our synthesized metal complexes showed better inhibition properties against MCF7 cancer cell lines. In addition, they showed that apoptotic bodies lead to cell death, as proved by variations such as restriction of cell growth, cytoplasmic condensation, and loss of membrane integrity^70^.

### Analyses of DNA fragmentation and apoptosis against MCF7 and HeLa cancer cell lines using SBLPr and SBLEr metal complexes

**Figure 9:**
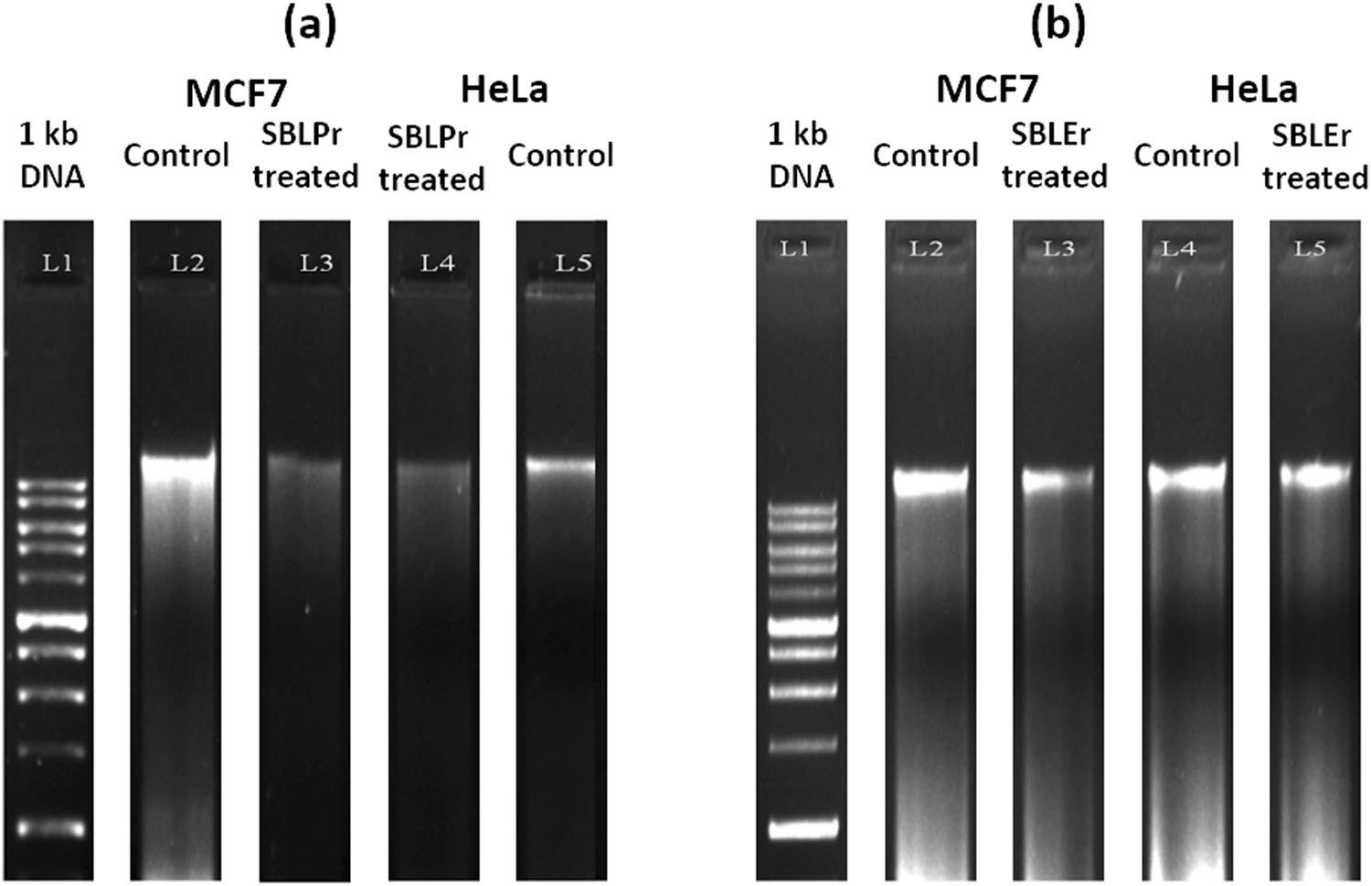
(**a**) DNA fragmentation of MCF7 and HeLa IC_50_ cells treated with SBLPr complex at 24 h. Lane 1: 1 kb DNA ladder, Lane 2: MCF7 control DNA, Lane 3: SBLPr-treated MCF7 cell (25 μg/ml), Lane 4: SBLPr-treated HeLa cell (25 μg/ml), Lane 5: HeLa control DNA. (**b**) DNA fragmentation of MCF7 and HeLa IC_50_ cells treated with SBLEr complex at 24 h. Lane 1: 1 kb DNA ladder, Lane 2: MCF7 control DNA, Lane 3: SBLEr-treated MCF7 cell (25 μg/ml), Lane 4: HeLa control DNA, Lane 5: SBLEr-treated HeLa cell (25 μg/ml).

DNA fragmentation is extensively assumed a distinctive of apoptosis^68,71^. The initiation of apoptosis may be established by irregular deficiency in cell size, in which the cells are condensed, and DNA fragmentation^72^. The observation of oligonucleosomal-shaped rubbles from the chromosomal DNA cleavage in is a primary part of apoptosis. Sophisticated biochemistry work has recognized the fragmentation factor of DNA leading apoptotic endonucleases in the destruction of DNA *in vitro*. Numerous reports are available on the capability of metal complexes for catalyzing the cleavage of DNA^70^. We therefore examined DNA-cleaving activities by an electrophoresis assay using SBLPr and SBLEr complex-examined MCF7 and HeLa cells. In the present study, HeLa and MCF7 IC_50_ cells were treated with SBLPr and SBLEr for 24 h, showing a decrease in cell survival by including DNA fragmentation. Figure 9(a,b) indicates the induction of apoptosis in the intermediary smears. The full-length electrophoresis images are presented in Supplementary Figure S2. For comparison, untreated control chambers are provided to explore the observation of no DNA fragmentation.

**Figure 10:**
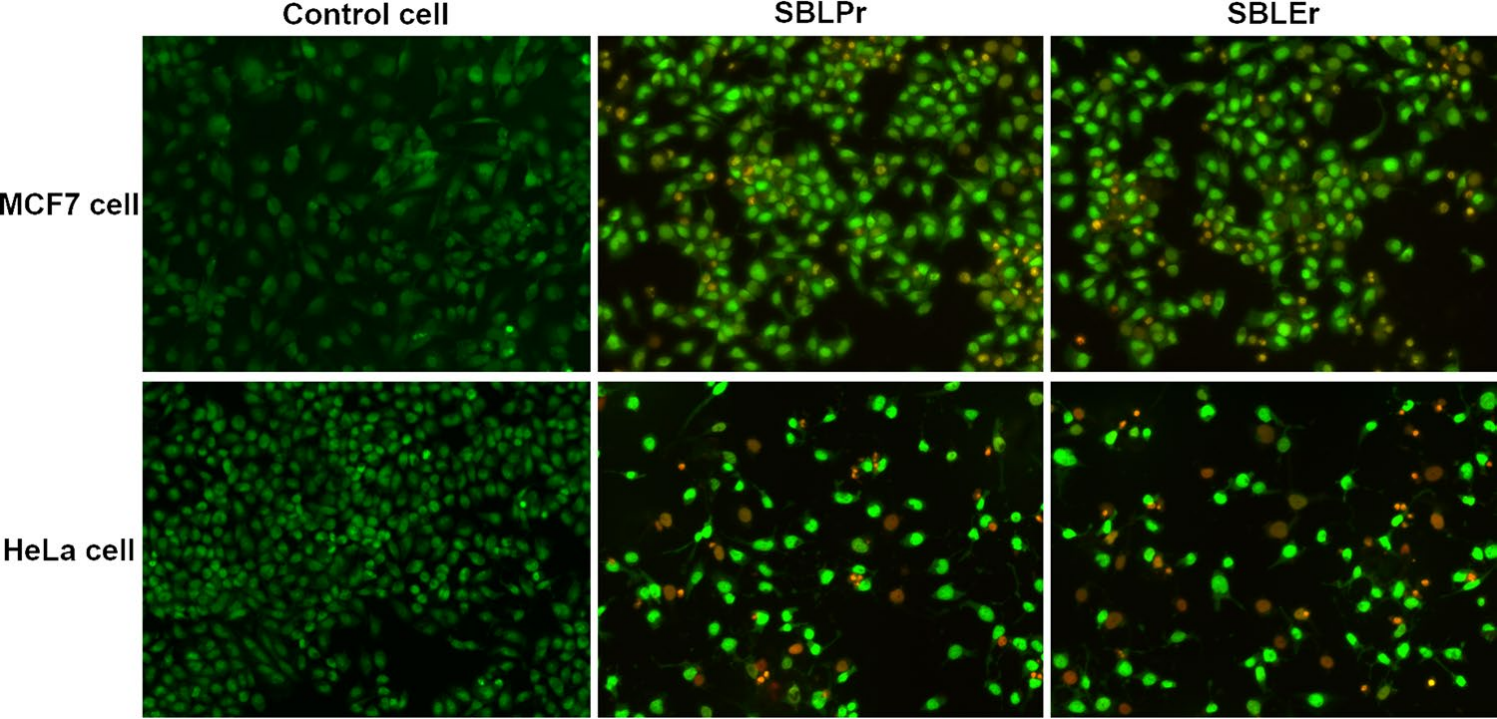
Morphological variations of AO/EB double-stained MCF7 and HeLa cells and their SBLPr- and SBLEr-treated cells using 25 μg/ml for 24 h.

**Figure 11:**
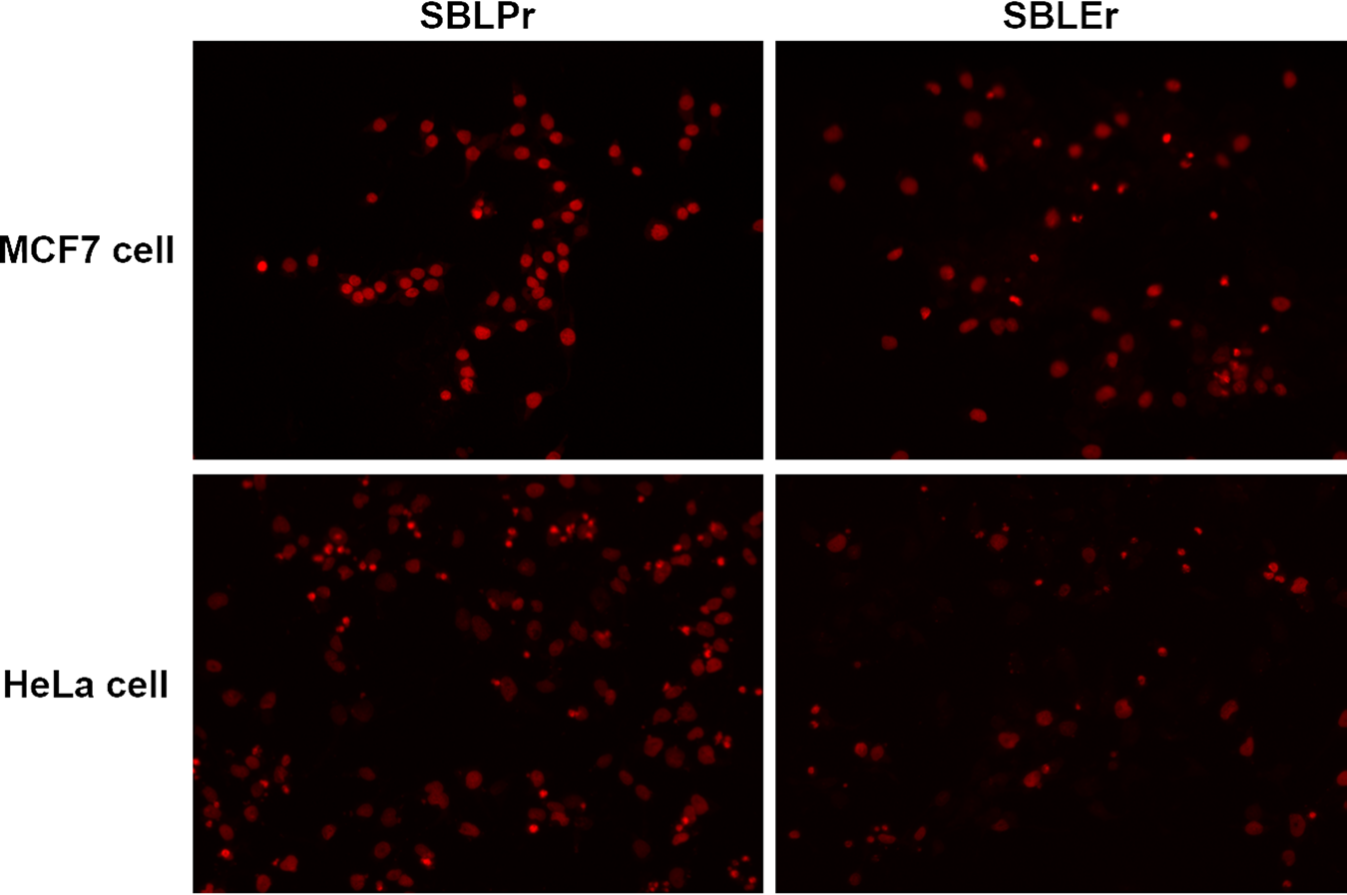
Morphological variations of PI-stained MCF7 and HeLa cells after treated with SBLPr and SBLEr using 25 μg/ml for 24 h.

Most of the small-molecule anticancer drugs employ their cytotoxic effects via apoptosis, which is monitored by the acridine orange (AO)/ethidium bromide (EB) dual staining method. As shown in Fig. 10, SBLPr and SBLEr efficiently induced apoptosis in MCF7 and HeLa cells. Apoptosis is commonly characterized by different morphological features, such as nuclear fragmentation, chromatin condensation, or the formation of apoptotic bodies^73,74^. To gain more evidence of the induction of apoptotic cell death by the SBLPr and SBLEr complexes, the apoptosis-inducing properties of MCF7 and HeLa cells were further examined by fluorescence microscopy using propidium iodide (PI) staining. In the case of MCF7 and HeLa IC_50_ cells treated with SBLPr and SBLEr, at 24 h, they displayed a progressive growth in the number of PI-positive cells. As shown in Fig. 11, nuclear fragmentation, chromatin condensation, and the formation of apoptotic bodies occurred following the treatment of MCF7 and HeLa cells with 25 μg/mL SBLPr and SBLEr complexes for 24 h. The results of the cell cycle and apoptosis experiments demonstrate that SBLPr and SBLEr complexes can effectively induce DNA damage and thus lead to cell cycle arrest and apoptosis in MCF7 and HeLa cells. Similarly, in an earlier study, curcumin- or catechin-examined different cell lines (HCT15, HCT116, and HepG2) showed the enrichment of condensed nuclear morphology, chromatin fluorescence, and the existence of dead cells^43^. This suggests that SBLPr and SBLEr metal complexes are stimulated the cell death in MCF7 and HeLa cells by the reactive oxygen species (ROS)-imposed apoptotic process. The enrichment of ROS levels and consequent damage of mitochondria membrane potency could be enhanced the cell death effectively.

## Conclusion

The development of an intense method to synthesize an SBL and its novel lanthanide metal complexes was demonstrated by simple one-pot chemical synthesis. The FT-IR spectral studies revealed that the SBL performed as a neutral bidentate ligand and it was bound with metal ions via the two azomethine nitrogens. In the electrophoresis experiments, SBLPr and SBLEr complexes were found to cause significant DNA cleavage of MCF7 and HeLa cells. In MTT assay cytotoxicity studies, SBLPr and SBLEr complexes exhibited anticancer activity against Vero, MCF7, and HeLa cancer cells. From the results of AO/EB staining assay, SBLPr and SBLEr complexes induced the apoptosis of MCF7 and HeLa cells. The PI staining assay showed that SBLPr and SBLEr complexes indeed induced DNA fragmentation, which provides additional confirmation of apoptosis. We hope that the observed results clearly demonstrate the cytotoxicity effects of SBLPr and SBLEr complexes against human cancer cell lines and that they become novel antitumor agents for humans.

## Experimental Details

### Synthesis of SBLs

A 2,3-diaminopyridine (10 mmol) solution was mixed with a 9-anthraldehyde (10 mmol) solution in a 1:2 molar ratio in methanol medium. The mixed solution was continuously stirred using magnetic stirrer for 2 h at 80 °C. Then, it was allowed to cool to room temperature, and the yellowish-orange crystalline product was separated out. It was then washed with ice-cold methanol and dried. The purity of the compound was examined with thin-layer chromatography. The collected yield was as high as 80%.

### Synthesis of lanthanide–SBL complexes

The lanthanide–SBL complexes were synthesized as per the following standard procedure. The SBL (0.2 mmol) was dissolved in methanol. Then, 0.1 mmol of praseodymium nitrate as a source for lanthanide was slowly added to the above solution. The reaction mixture was refluxed in an oil bath at 80 °C for 4 h. After the specific period, the solution was allowed to cool gradually, and the SBL metal complex was collected in the liquid phase. Then, it was filtered using Whatman filter paper. The collected SBL metal complex was washed with diethyl ether and dried in a vacuum oven. The same procedure was repeated using erbium nitrate and ytterbium nitrate instead of praseodymium nitrate to prepare the Yb- and Er-based SBL complexes. The crude precipitate of [Pr (Er or Yb) (NO_3_)_2_(H_2_O)], NO_3_ was purified from the acetonitrile-methanol mixture.

### Anticancer activities of SBL–lanthanide complexes

#### Cell culture

All the cancer cell lines such as Vero, MCF7 (breast cancer), and HeLa (cervical cancer) were purchased from the National Centre for Cell Sciences (NCCS), Pune, India. The purchased cells were preserved in the logarithmic level of growth in DMEM medium accompanied with 10% (v/v) heat-inactivated fetal bovine serum, 100 µg/mL penicillin, and 100 µg/mL streptomycin. Then, the samples were preserved at 37 °C in a 5% CO_2_–95% air humidified incubator. The cytotoxic effects of the SBL and its Pr and Er complexes were tested against cancer cell lines (i.e., Vero, MCF7, and HeLa) by MTT (3-(4,5- dimethylthiazol-2-yl)−2,5-diphenyltetrazolium bromide assay^75^.

#### Cytotoxicity analysis against cancer cell lines

The sustainable Vero, HeLa, and MCF7 cells were individually grown in 96-well microplates (1 × 10^6^ cells /mL) and incubated at 37 °C for 24 h with a 5% CO_2_ incubator to allow them to develop to 90% confluence. Thereafter, the medium was exchanged, and the Vero cells were examined with SBLPr and SBLEr at various concentrations of 5, 10, 25, 50, 75, and 100 µg/mL. Then, the samples were incubated for 24 h. The samples were take-out from the incubator and washed with phosphate-buffered saline (PBS, pH- 7.4). Then, 20 μL of MTT solution (5 mg/mL) was mixed to each well, and they were performed to stand at 37 °C in the dark for an extra 4 h. Then, 100 μL of DMSO was added, and the formazan crystals were dissolved. The absorbance was measured spectrometrically at 570 nm using an ELISA plate reader. The percentage of cell viability was estimated as follows:

1 $$Cell\,viability( \% )=(Absorbance\,of\,treated\,cells/Absorbance\,of\,control\,cells)\times {100}$$

The 50% of inhibited cell growth was denoted as the IC_50_ value, which was used as a parameter for the cytotoxicity study. The morphological changes of untreated (control) and examined IC_50_ cells were observed after 24 h under a bright field microscope. The cytotoxicity results were received from three independent measurements by different cell passages for all the cancer cell lines, although each measurement was implemented in triplicate.

#### Assessment of apoptosis

HeLa and MCF7 cells were separately plated at a density of 5 × 10^4^ cells/well into a 6-well chamber plate. At >90% confluence, the cells were treated with SBLPr and SBLEr complexes for 24 h. The cells were washed with PBS fixed in methanol: acetic acid (3:1, v/v) for 10 min and stained with 50 µg/mL PI for 20 min. For the nuclear analysis, the cell plates were washed with PBS and stained with 5 μL of AO (100 μg/mL) and 5 μL of EB (100 μg/mL). The morphological variations in the stained cells, including apoptotic nuclei (condensed chromatin, fragmented nuclei, and intensely stained), were perceived by fluorescence microscopy. Further, electrophoresis analysis were carried out for HeLa and MCF7 cell lines using a get tank consisted of ethidium bromide with one percentage of agarose gel at 1 hour in 90 V by reported method^68,76^.

### Characterization

The synthesized SBL and its rare earth metal complexes were characterized by UV-vis spectroscopy using a Perkin Elmer LS25 spectrophotometer with methanol as a solvent. FT-IR analyses were carried out using a JASCO spectrophotometer. The ^1^H NMR spectrum was recorded using a Bruker Advance DPZ-300 spectrometer operating at 300 MHz using DMSO (spectroscopic grade) as a solvent and TMS as an internal standard. The surface morphology of SBL and its metal complexes was analyzed by FESEM (FEI Quanta 250 Czech Republic). Fluorescence spectra were measured by using a Perkin Elmer LS45 fluorescence spectrophotometer. The thermal analysis was performed on a PCT-2A thermo balance analyzer operating at a heating rate of 10 °C/min in the range of ambient temperature to 720 °C under N_2_. The molar conductance study was carried out using conductivity meter instrument (model CM Elico-185) with 1 mM DMSO as a solvent at room temperature.

## Electronic supplementary material

Supporting information
