# Supplementary material for: In vitro cytotoxicity activity of novel Schiff base ligand–lanthanide complexes
Source: Sci Rep. 2018 Feb 14;8:3054. doi: 10.1038/s41598-018-21366-1 (PMC5812993; doi:10.1038/s41598-018-21366-1)
Supplement: Supplementary file 1 — Supporting information [file 41598_2018_21366_MOESM1_ESM.doc]

Supporting Information

***In vitro* cytotoxicity activity of novel Schiff base ligand–lanthanide complexes**

Kavitha Andiappan^a,b^, Anandhavelu Sanmugam^a^, Easwaramoorthy Deivanayagam^b*^, K. Karuppasamy^c^, Hyun-Seok Kim^c*^, Dhanasekaran Vikraman^c*^

^a^Department of Chemistry (S & H), Vel Tech Multi Tech., Chennai – 600 062, India.

^b^Department of Chemistry, B.S Abdur Rahman University, Vandalur, Chennai-600048, India.

^c^Division of Electronics and Electrical Engineering, Dongguk University-Seoul, Seoul 04620, Republic of Korea.

*Corresponding authors’ E-mail: [v.j.dhanasekaran@gmail.com](mailto:v.j.dhanasekaran@gmail.com) (Dhanasekaran Vikraman), [hyunseokk@dongguk.edu](mailto:hyunseokk@dongguk.edu) (Hyun-Seok Kim) and [easwar@bsauniv.ac.in](mailto:easwar@bsauniv.ac.in) (Easwaramoorthy Deivanayagam)

**Table S1. UV-Vis absorption peaks for SBL and its metal complexes**

| **Compound** | **Absorption (nm)** | **Band assignment** | **Geometry** |
| --- | --- | --- | --- |
| SBL | 276  313 | INCT  INCT | ---  --- |
| SBLPr | 276  313  442 | INCT  INCT  ^2^B_1g_→^2^A_1g_ | ---  ---  Distorted Square planar |
| SBLEr | 268  312  445 | INCT  INCT  ^2^B_1g_→^2^A_1g_ | ---  ---  Distorted Square planar |
| SBLYb | 277  310  450 | INCT  INCT  ^2^B_1g_→^2^A_1g_ | INCT  INCT  ^2^B_1g_→^2^A_1g_ |

**Table S2. The important FT-IR bands for SBL and its metal complexes**

| **Compound** | **ν_C-H_ (cm^-1^)** | **ν_HC=N_ (cm^-1^)** | **ν_M-N_**  **(cm^-1^)** | | **ν_phenyl ring N-atom_ (cm^-1^)** | **ν_phenyl ring_**  **(cm^-1^)** |
| --- | --- | --- | --- | --- | --- | --- |
| SBL | 2853 | 1608 | --- | ---- | 508 | 1108 |
| SBLPr | 2852 | 1661 | 456 | 426 | 503 | 1116 |
| SBLEr | 2850 | 1660 | 454 | 425 | 502 | 1113 |
| SBLYb | 2846 | 1662 | 458 | 426 | 500 | 1118 |

**Table S3: Molar conductance value for SBL and its metal complexes**

| **Compound** | **Color** | **Molar conductance**  **λ_M_  (Ω^-1^ cm^2^ mol^-1^)** |
| --- | --- | --- |
| SBL | Yellow | **---** |
| SBL-Pr | Orange | 108 |
| SBL-Er | Yellow color | 117 |
| SBL-Yb | Light yellow | 128 |

**Table S4. TG-DTA weight loss and residue details for SBL and its metal complexes**

| **Compound** | **1^st^ Stage, ºC (wt. loss %)** | **2^nd^ Stage, ºC (wt. loss %)** | **3^rd^ Stage, ºC (wt. loss %)** | **4^th^ Stage, ºC (wt. loss %)** | **Residue %**  **(at 750 ºC)** |
| --- | --- | --- | --- | --- | --- |
| SBL | 237-345, (1.2) | 350-557, (59.8) | 601-659, (28) | ----- | 11 |
| SBL-Pr | 30-120, (6) | 226-349, (20.8) | 365-453, (22) | 461-630,  (31.2) | 20 |
| SBL-Er | 30-100, (10) | 136-425, (22) | 452-570, (32) | 583-650, (10) | 26 |
| SBL-Yb | 30-124, (1) | 170-327, (6) | 376-493, (35) | 495-632, (25) | 33 |


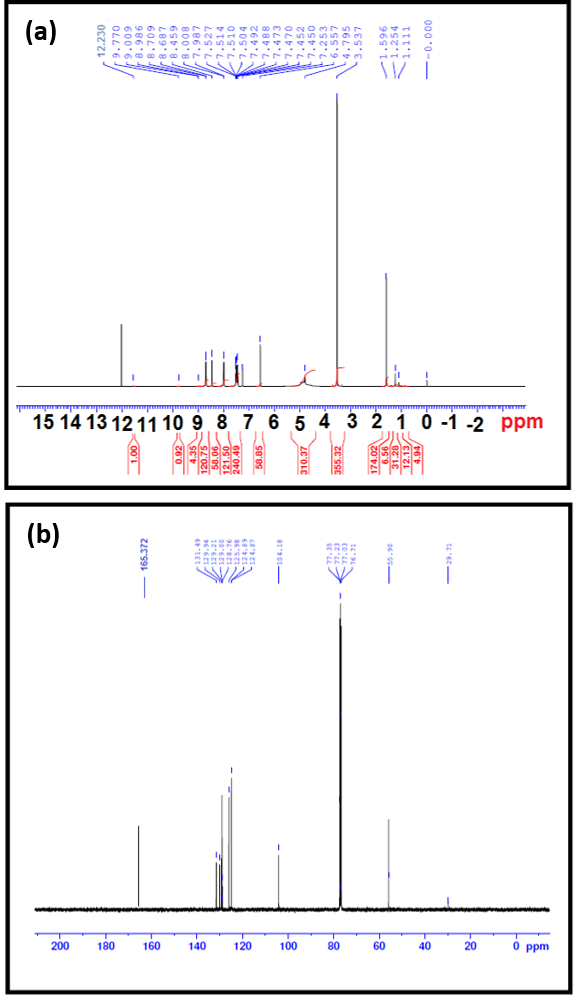


**Figure S1 (a) ^1^H and (b) ^13^C NMR spectra of SBL**

**
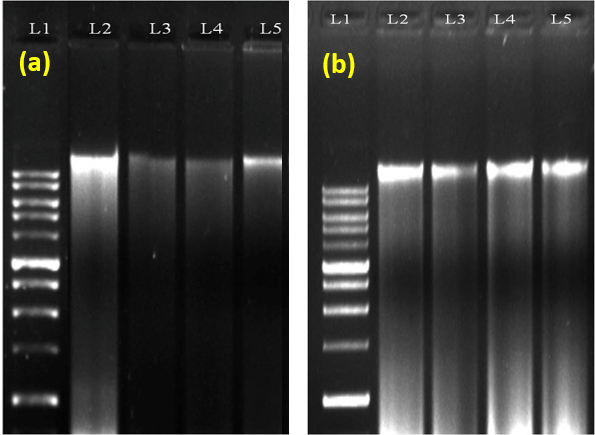
**

**Figure S2.** **DNA fragmentation of MCF7 and HeLa IC_50_ cells treated with (a) SBLPr and (b) SBLEr metal complexes at 24 h.**
